# Toxicities Associated with Cisplatin-Based Chemotherapy and Radiotherapy in Long-Term Testicular Cancer Survivors

**DOI:** 10.1155/2018/8671832

**Published:** 2018-02-18

**Authors:** Chunkit Fung, Paul Dinh, Shirin Ardeshir-Rouhani-Fard, Kerry Schaffer, Sophie D. Fossa, Lois B. Travis

**Affiliations:** ^1^James P. Wilmot Cancer Institute, University of Rochester Medical Center, Rochester, NY, USA; ^2^Melvin and Bren Simon Cancer Center, Indiana University, Indianapolis, IN, USA; ^3^Department of Oncology, Oslo University Hospital, Radium Hospital, Oslo, Norway

## Abstract

Testicular cancer has become the paradigm of adult-onset cancer survivorship, due to the young age at diagnosis and 10-year relative survival of 95%. This clinical review presents the current status of various treatment-related complications experienced by long-term testicular cancer survivors (TCS) free of disease for 5 or more years after primary treatment. Cardiovascular disease and second malignant neoplasms represent the most common potentially life-threatening late effects. Other long-term adverse outcomes include neuro- and ototoxicity, pulmonary complications, nephrotoxicity, hypogonadism, infertility, and avascular necrosis. Future research efforts should focus on delineation of the genetic underpinning of these long-term toxicities to understand their biologic basis and etiopathogenetic pathways, with the goal of developing targeted prevention and intervention strategies to optimize risk-based care and minimize chronic morbidities. In the interim, health care providers should advise TCS to adhere to national guidelines for the management of cardiovascular disease risk factors, as well as to adopt behaviors consistent with a healthy lifestyle, including smoking cessation, a balanced diet, and a moderate to vigorous intensity exercise program. TCS should also follow national guidelines for cancer screening as currently applied to the general population.

## 1. Introduction

Testicular cancer (TC) is the most common cancer, affecting young men aged 18–39 years [1]. Due to effective cisplatin-based chemotherapy introduced in the 1970s [2], TC is highly curable with a 10-year relative survival approaching 95% [3, 4]. However, treatment-related complications, including cardiovascular disease (CVD), second malignant neoplasms (SMN), neuro- and ototoxicity, pulmonary complications, nephrotoxicity, hypogonadism, infertility, avascular necrosis, cognitive impairment, anxiety/depression, and chronic cancer-related fatigue, accompany these remarkable successes [5–7]. These adverse outcomes of TC and its therapy have emerged as important issues for this young cohort of survivors. In this review article, we will focus on toxicities due to cisplatin-based chemotherapy and radiotherapy experienced by long-term survivors of TC, which are defined as individuals who are disease-free 5 years or more after primary treatment [8]. Due to sparse data, the risks of long-term toxicities after single-dose carboplatin for stage I seminoma or one to two cycles of bleomycin, etoposide, and cisplatin (BEP) for stage I nonseminoma will not be reviewed.

## 2. Cardiovascular Disease and Raynaud Phenomenon

A few hypotheses have been proposed to explain the pathophysiology of CVD in TC survivors (TCS), including the direct vascular damage hypothesis, the indirect hypothesis, and more recently the multiple-hit hypothesis [9, 10]. The direct vascular damage hypothesis proposes that cisplatin-based chemotherapy causes direct damage to the vascular endothelium [9]. In vitro exposure of endothelial cells to cisplatin or bleomycin causes cytokine release and cytotoxicity [11, 12]. Von Willebrand factor, an inflammatory marker released by endothelial cells in response to vascular damage, increases in TC patients during chemotherapy [13]. Other markers of inflammation and endothelial dysfunction are also evident after cisplatin-based chemotherapy, including fibrinogen, tissue-type plasminogen activator, and high-sensitivity C-reactive protein [14, 15]. Microalbuminuria is present in an increased number of TC patients treated with cisplatin-based chemotherapy [14, 16], which is a clinical manifestation of systemic vascular dysfunction that independently predicts for vascular events, including stroke and myocardial infarction (MI) [17]. In one study, microalbuminuria persisted in 22% of TCS treated with cisplatin-based chemotherapy after a median follow-up of 14 years [16].

A prior investigation [15] showed that the carotid intimal medial thickness of TC patients, which correlates with increased risk of cerebrovascular accidents and MI [18], significantly increased during a 3.5-month course of cisplatin-based chemotherapy. This rate of increase was significantly higher than the annual change observed in carotid intimal medial thickness in the general population. Acute alterations in diastolic heart function were reported in a study [19] of 14 TC patients three months after initiation of 3 to 4 cycles of chemotherapy with BEP; these included significant decreases in the left ventricular end-diastolic and stroke volumes. Other suggested mechanisms of direct vascular damage include cisplatin-induced vasospasm due to hypomagnesemia [20–23] and increased formation of procoagulant endothelial microparticles released by endothelial cells, triggering thrombin generation and hypercoagulability [24, 25].

Raynaud phenomenon is another clinical manifestation of vascular damage and is estimated to be present in approximately 25% to 61% of TCS [26–30]. The onset of symptoms from Raynaud phenomenon generally begins within 4 to 12 months of chemotherapy, with 25% experiencing these symptoms up to 20 years [14]. Bleomycin is strongly associated with the development of Raynaud phenomenon. In a randomized study [31] of 395 patients with good-risk metastatic nonseminoma, 8% of patients randomized to BEP developed Raynaud phenomenon compared to none undergoing etoposide and cisplatin (EP). Vinblastine and cisplatin are other chemotherapeutic agents that may contribute to this toxicity [28–30, 32].

The indirect hypothesis postulates that cisplatin-based chemotherapy increases the prevalence of CVD risk factors in TCS, resulting in increased CVD events [9]. Multiple studies [14, 16, 30, 33–40] have reported increased frequency of hyperlipidemia, hypertension, diabetes, insulin resistance, and metabolic syndrome among TC patients after treatment with chemotherapy compared to surgery-only comparison groups or controls derived from the general population (Table 1). Although several studies [36, 38, 41] showed that metabolic syndrome and its individual components are associated with testosterone deficiency and hypogonadism, most TCS with CVD risk factors have normal testosterone levels [33]. Decreased testosterone levels may cause endothelial dysfunction, impair vascular smooth muscle reactivity, increase intima and media thickness of vessels, and increase synthesis of proinflammatory cytokines [42–44]. In an investigation by Haugnes et al. [33], relationships with both hypogonadism and cumulative dose of cisplatin and metabolic syndrome were evaluated among 1135 Norwegian TCS. Compared to the surgery group, TCS who received a cumulative dose of cisplatin >850 mg had a significant 2.8-fold increased odds of metabolic syndrome, with both total serum testosterone and smoking history (≥20 pack-years) being independent predictive factors in multiple regression models.

A multiple-hit hypothesis that encompasses both the direct and indirect hypotheses has recently been proposed to explain the elevated risk of CVD among TCS [10, 45]. This model hypothesizes that multiple factors interact synergistically to increase the risks of CVD among TCS, including orchiectomy-derived subclinical hypogonadism, chemotherapy-induced vascular injury, chemotherapy-related disturbance of metabolic homeostasis, and other TC treatment-related toxicities [10].

The relative risk of CVD among TCS treated with chemotherapy is 1.4- to 7.1-fold significantly higher compared to the general population or to those managed with surveillance only [16, 34, 35, 46, 47]. A British study [35] of 390 TCS treated with chemotherapy between 1982 and 1992 at a median follow-up of 9.7 years showed a 7% incidence of angina, MI or sudden cardiac death, with an elevated age-adjusted relative risk (RR) of 2.6 (95% confidence interval (CI) 1.2–5.8) when compared with TC patients treated with surgery alone. In a retrospective study [46] of a nationwide cohort of 2707 5-year TCS in the Netherlands (1965–1995) after a median follow-up of 17.6 years, cisplatin-based chemotherapy (cisplatin, vinblastine, bleomycin (PVB) or BEP) increased the risk of CVD by 1.7-fold (95% CI 1.1–2.5) when compared with age and sex-matched data in the general Dutch population.

To determine CVD risk after modern-era cisplatin-based chemotherapy in TC patients, Haugnes et al. [34] evaluated the prevalence of cardiovascular risk factors and long-term incidence of CVD among 990 5-year TC survivors (median follow-up: 19 years). All cytotoxic treatment groups (radiation only, chemotherapy only, and combined radiation/chemotherapy) had significantly increased prevalence of usage of antihypertensive medications compared with age-matched male controls in the general population. The odds of diabetes were higher in the radiation (odds ratio (OR) 2.3; 95% CI 1.5–3.7) and radiation/chemotherapy groups (OR 3.9; 95% CI 1.4–10.9) compared to controls [34]. Using age-adjusted Cox regression analyses, increased risks of atherosclerotic disease were reported in the radiation only (hazard ratio (HR) 2.3; 95% CI 1.04–5.3), chemotherapy only (HR 2.6; 95% CI 1.1–5.9), and combined radiation/chemotherapy cohorts (HR 4.8; 95% CI 1.6–14.4) compared to those managed with surgery only [34]. Treatment with BEP alone increased the risk of coronary artery disease by 5.7-fold (95% CI 1.9–17.1) compared with surgery only, while the risk for MI increased by 3.1-fold (95% CI 1.2–7.7) compared with age-matched male controls [34].

Using age-adjusted Cox regression analyses, increased risks of atherosclerotic disease were reported after radiation only (HR 2.3; 95% CI 1.04–5.3), chemotherapy only (HR 2.6; 95% CI 1.1–5.9), and combined radiation/chemotherapy (HR 4.8; 95% CI 1.6–14.4) compared with surgery only (P-trend = 0.02) [34]. In particular, treatment with BEP alone increased CAD risk by 5.7-fold (95% CI 1.9–17.1) compared with surgery only and increased MI risk by 3.1-fold (95% CI 1.2–7.7) compared with age-matched male controls [34].

Several studies [48–50] have examined the extent to which increased CVD mortality might result from TC treatment. In an international population-based study [48] of 38,907 TCS (1943–2002) at a median follow-up of 10 years, a 1.6-fold (95% CI 1.3–2.0) increased risk of mortality from all circulatory diseases was reported for those treated with chemotherapy after 1975. Another population-based study [49] using the SEER program (1973–2008) found that patients with either mediastinal or nonmediastinal extragonadal GCT had significantly increased 4.5-fold and 2.8-fold risks of CVD mortality, respectively, compared to patients with primary testicular GCT. The increased number of cycles of primary chemotherapy and additional salvage chemotherapy typically required to treat extragonadal TC were hypothesized to contribute to this higher risk, although detailed chemotherapy data were not available [49]. Recently, Fung et al. [50] reported a significant 5.3-fold increase in CVD mortality during the first year after chemotherapy in a population-based study of 15,006 TCS managed initially with either chemotherapy or surgery alone without radiotherapy during 1980–2010. In contrast, excess CVD mortality was not observed more than one year after chemotherapy, likely due to advances in cardiovascular disease management, as reflected in the 31% decline in US cardiovascular death rates from 2000 to 2010 [51]. In multivariable analyses, increased CVD mortality after chemotherapy was confined to the first year after TC diagnosis (HR 4.86; 95% CI, 1.25–32); distant disease (*P* < 0.05) and older age at diagnosis (*P* < 0.01) were independent risk factors [50].

Currently, there are no established evidence-based CVD screening recommendations developed specifically for TCS. In November 2013, the American College of Cardiology and the American Heart Association released guidelines for the assessment of cardiovascular disease risk, the management of elevated cholesterol and increased body weight, and lifestyle modifications to reduce CVD risk in adults in the general population [52]. Health care professionals should monitor and modify cardiovascular risk factors of TCS by referring to these guidelines [52] and by leveraging TC diagnosis as a teachable moment to promote lifestyle changes, including smoking cessation, optimal nutrition, and a nonsedentary lifestyle [7, 53].

## 3. Second Malignant Neoplasms

**Figure 1:**
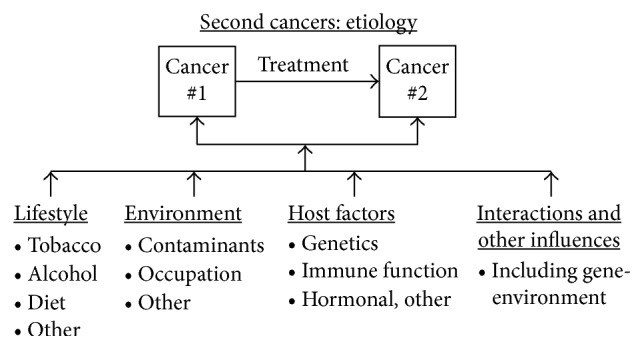
Risk factors for second primary cancer (refer to text). Many influences some of which are diagrammed here may contribute to the development of multiple primary cancers, including interactions between exposures. ^∗^Adapted with permission from Travis [169].

Syndromic, cancer treatment, and shared etiologic exposures are the major causative factors of SMN [54].  Figure 1 shows the influence of lifestyle factors, genetic susceptibility, environmental exposures, host effects, and a combination of influences, including gene-environment interactions in the development of SMN. Age at exposure and attained age are modifiers for the risks of selected SMN [55].

After receiving radiotherapy for TC treatment, TCS have significantly increased risks of leukemia [56] and solid cancers [46, 55, 57–59] (Table 2). An international population-based study of 18,567 TCS reported a significantly 3-fold increased risk of leukemia after abdominal and pelvic radiotherapy with a mean dose of 10.9 Gy to active bone marrow [56]. The median latency for leukemia was 5.0 years with a quarter of survivors developing leukemia more than one decade later (maximum latency: 17.3 years) [56]. After radiation treatment, long-term TCS also have significantly 1.4 to 1.9-fold increased risks of second solid cancers compared to the general population (Table 2) [46, 55, 57]. An international population-based investigation of 10-year TCS reported that the RR of SMN at sites included in typical infradiaphragmatic radiotherapy fields were significantly larger than risks at unexposed sites (RR 2.7 versus 1.6; *P* < 0.05), which remained elevated for more than 35 years. In another study [46], infradiaphragmatic radiotherapy administered at doses 40–50 Gray (Gy) compared with 26–35 Gy increased the HR for SMN from 2.3 to 3.2, respectively, when using a surgery-only group as control. Two recent studies of 5-year TCS reported a 5.9-fold increased risk of stomach cancer (95% CI 1.7–20.7) [58] and a 2.9-fold increased risk of pancreatic cancer (95% CI 1.0–7.8) after radiotherapy [59]. The risks of stomach and pancreatic cancers increased with higher radiation doses to stomach [58] and pancreas [59], respectively (*P* trend < 0.001), and risks remained elevated for ≥20 years after exposure (*P* < 0.01) [58, 59]. Several other studies of TCS [46, 60, 61] similarly reported significant associations between radiotherapy and SMN risks.

Cisplatin and etoposide are integral chemotherapeutic agents used in standard chemotherapy regimens to treat TC [62]. Both cisplatin and etoposide are associated with significantly elevated risks of secondary leukemia [56, 63–65]. An international nested case-control study [56] among TCS estimated a 3.2-fold risk of leukemia after cumulative cisplatin dose of 650 mg, although the excess risk was small with only 16 excess cases among 10,000 TC patients after 15 years of follow-up. The same study also reported a significant dose-response relationship between cumulative dose of cisplatin and leukemia risk after adjustment for radiation dose (*P* trend = 0.001) [56]. The 5-year cumulative incidence of leukemia is approximately 0.5% after a cumulative etoposide dose of <2000 mg/m^2^ and 2.0% after a cumulative etoposide dose of ≥2000 mg/m^2^ [65].

Most prior studies of second solid cancer focused on TCS treated before modern cisplatin-based chemotherapy became widely adopted prior to early 1980s (Table 2) [10, 13, 22, 23]. Whereas an international series of more than 40,000 TCS showed a 1.8-fold (95% CI 1.3–2.5) significantly increased risk of second solid cancers among a subgroup of 10-year TCS who received initial chemotherapy during 1943–2001, three smaller epidemiologic studies [10, 39, 57] (ranging from 346 to 710 patients) found no significantly elevated risk of SMN after chemotherapy [27, 46, 66], though they may have inadequate statistical power. To evaluate the risks of second solid cancer among TCS treated in the modern era of cisplatin-based chemotherapy during 1980 to 2008, a recent large population-based investigation by Fung et al. [67] of more than 12,000 TCS reported a 1.4-fold significantly increased risk of solid cancers after initial treatment with chemotherapy compared to those who underwent initial surgery alone. Significantly increased three- to seven-fold risks of cancers of the kidney (standardized incidence ratio (SIR) 3.4), thyroid (SIR 4.4), and soft tissue (SIR 7.5) were also observed. After chemotherapy, elevated risks of solid cancer were reported in most follow-up periods with a median latency of 12.5 years, including at more than 20 years after treatment (SIR 1.54; 95% CI 0.96–2.3). However, detailed information on cytotoxic drug name and dose were not available [67].

TCS should follow national guidelines for cancer screening as applied to the general population, given their increased risks of SMN [53]. Earlier or additional cancer screening may be clinically indicated in TCS deemed at high risk due to prior treatment history and/or health habits [53]. In addition, health care providers should advise TCS of the modest 15-year cumulative risk (1.9%) of metachronous contralateral testicular cancer [68].

## 4. Neurotoxicity

Approximately 20 to 40% of long-term TCS experience symptoms of peripheral neuropathy after cisplatin-based chemotherapy [28, 29, 69]. Common clinical manifestations of peripheral neuropathy include numbness, tingling, and a decrease in vibratory sense in distal extremities [70]. The cumulative dose of cisplatin administered affects the incidence of peripheral neuropathy. At a median of 11 years after TC treatment, 46% of TCS in a population-based long-term Norwegian survey self-reported paresthesia after ≥5 cycles of cisplatin-based chemotherapy compared to 28% after 1 to 4 cycles of chemotherapy or 10% after orchiectomy alone [28]. Compared to TCS who did not receive chemotherapy, those who underwent 1 to 4 cycles and ≥5 cycles of cisplatin-based chemotherapy had higher risks of symptomatic paresthesia of the hands (OR 2.0, 95% CI 1.5–2.7; OR 3.9, 95% CI 2.1–7.3, resp.) and feet (OR 2.2, 95% CI 1.7–3.0; OR 3.1, 95% CI 1.7–5.7, resp.) [28]. In the same study [28], radiotherapy was significantly associated with symptomatic paresthesias of the feet (OR 1.5), but retroperitoneal lymph node dissection (RPLND) was not an independent risk factor. Increasing levels of residual serum platinum are also directly associated with severity of neurotoxicity after adjusting for initial cisplatin dose [71]. Sprauten et al. reported [71] that the total score for the Scale for Chemotherapy Induced Neuropathy (SCIN) had a significant four-to five-fold association with the highest residual serum platinum quartile in cisplatin-treated TC patients.

Oldenburg et al. [72] investigated the impact of germline single nucleotide polymorphisms (SNPs) of glutathione S-transferase (*GST*) *P*1, *M*1, and *T*1 on self-reported paresthesia among long-term TCS. The *GSTP1-GG* genotype conferred a significantly lower risk of developing paresthesia in the fingers (OR = 0.46, 95% CI 0.22–0.96) and toes (OR = 0.42, 95% CI 0.20–0.88) than the *GSTP1-AA* and *GSTP1-AG* genotypes. Recently, a genome-wide analysis of cisplatin-induced peripheral neuropathy in survivors of adult-onset cancer reported that genetically determined expression level of *RPRD1B* was associated with cisplatin-induced peripheral neuropathy [73]. Defects in *RPRD1B* expression or knockdown cause a deficiency in DNA repair mechanisms known to be critical in repairing cisplatin-induced lesions [74] and result in increased sensitivity to cisplatin in a breast cancer cell line, MDA-123 [75].

No therapeutic agents are currently recommended for the prevention of peripheral neuropathy due to the paucity of high-quality, consistent evidence. For management of drug-induced peripheral neuropathy, the ASCO Clinical Practice Guideline [76] recommends treatment with duloxetine as potentially the most effective drug. Health care providers may also offer tricyclic antidepressants (i.e., nortriptyline), gabapentin, and a compounded topical gel containing baclofen, amitriptyline HCL, and ketamine based on clinical benefits observed for other neuropathic pain conditions [76].

## 5. Ototoxicity

Cisplatin selectively damages the outer hair cells of the cochlea [77], causing tinnitus and hearing loss that predominantly affect high frequencies [77–79] similar to age-related presbycusis. After a median follow-up of 58 months, Bokemeyer et al. [78] found that 20% of TCS (median age: 31 years) reported symptomatic ototoxicity (59% tinnitus, 18% hearing loss, 23% both) after cisplatin-based chemotherapy. For TCS who received >400 mg/m^2^ of cumulative cisplatin dose, 50% self-reported tinnitus and hearing loss compared to 20% of those treated with ≤400 mg/m^2^ [78]. Older age, higher cumulative cisplatin dose, a history of noise exposure, hypertension, and impaired baseline renal function and hearing are each independently associated with more severe ototoxicity [78–80]. A recent comprehensive audiometric analysis of 488 North American TCS [79] reported that almost one in five (18%) had severe to profound hearing loss as defined by the American Speech-Language-Hearing Association criteria (median follow-up: 4.25 years after completion of cisplatin-based chemotherapy). Tinnitus (40% patients) was significantly correlated with reduced hearing at each frequency (*P* < 0.001). The same study [79] also found that increasing cumulative cisplatin dose was significantly related to hearing loss at 4, 6, 8, 10, and 12 kHz (*P* trend for each <0.05). For each 100 mg/m [2] increase in cumulative cisplatin dose, a 3.2 dB impairment in age-adjusted overall hearing threshold (4–12 kHz; *P* < 0.001) resulted. However, cisplatin dose did not affect noise-induced hearing damage (10% patients) (*P*=0.59) [79].

A few reports have identified significant associations of germline genetic polymorphisms of various genes with platinum-related ototoxicity, including megalin [81], *GSTP1* [72, 82], *GSTM3* [72, 82], *COMT* [83], *TPMT* [83], and *WFS1* [84]. Both alleles of *105Val-GSTP1* protected against cisplatin-induced ototoxicity in cisplatin-treated TCS, whereas *GSTM1* positivity was detrimental for hearing ability [82]. Functional polymorphisms of the glutathione *S*-transferases (*GSTs*) genes likely cause differential expression of the cisplatin-detoxifying enzymes, consequently rendering TCS suspcetible to varying degrees of cisplatin-induced hearing impairment. A recent genome-wide association study [85] of 511 TC patients of European genetic ancestry reported that one SNP, rs62283056, in the first intron of *WFS1* (wolframin ER transmembrane glycoprotein) was significantly associated with cisplatin-associated ototoxicity (*P*=1.4 × 10^−8^), with higher cisplatin doses exacerbating hearing loss in TC patients with the risk allele. In the general population, *WSF1* mutations causes the Mendelian disorders DFNA6 (deafness, autosomal dominant 6) and the recessive Wolfram syndrome (with hearing loss) [86, 87].

There are no effective pharmacologic agents available to prevent or treat cisplatin-induced ototoxicity. TCS should use ear protection to minimize noise exposure and additional hearing loss. Since the peak concentration of cisplatin may be directly associated with the severity of ototoxicity [28, 88], where indicated, the 5-day BEP regimen seems preferable to a 3-day regimen [7].

## 6. Pulmonary Toxicity

The incidence of fatal bleomycin-induced pulmonary toxicity is approximately 1–3% [89, 90]. Corticosteroids remain the mainstay of treatment of bleomycin-induced pneumonitis, although there are no data from prospective randomized trials to support this approach [91]. Health care providers should withhold bleomycin at the earliest signs or symptoms of bleomycin-induced pulmonary toxicities during chemotherapy. Since age of more than 40 years [89] and increased tobacco use [92] are both significantly associated with pulmonary toxicity during bleomycin treatment, a careful assessment of patient history (i.e., age, smoking status, and preexisting lung disease) are important to consider prior to the administration of any bleomycin-containing regimen [93]. Avoiding perioperative overhydration is important to minimize the risk of perioperative lung complications, but perioperative oxygen restriction in patients a few months after administration of bleomycin is not necessary [94, 95].

Bleomycin hydrolase is an enzyme encoded by the *BLMH* gene, which inactivates bleomycin [96]. A Dutch investigation of 340 TC patients treated with bleomycin-containing chemotherapy between 1977 and 2003 reported that the genetic polymorphism of 1450A > G was not associated with bleomycin-induced pneumonitis or changes in pulmonary function tests [97].

A large Norwegian study [92] of 1049 long-term TCS treated during 1980 to 1994 (median follow-up: 11.2 years) reported that 8% of survivors had restrictive lung disease as defined by predicted FVC <80% and a value of ≥70% for forced expiratory volume (FEV) 1/forced vital capacity (FVC). In multivariate analyses adjusting for bleomycin, etoposide, and vinblastine doses, higher cumulative cisplatin dose (*P*=0.007) and older age (*P*=0.008) were both significantly related to restrictive lung disease [92]. Compared with men treated with surgery only, patients who received large cumulative doses of cisplatin (>850 mg) as well as combined chemotherapy and pulmonary surgery were at significantly increased risk of demonstrating decreased spirometry variables, including age-adjusted FVC, FEV1, FVC% predicted, and FEV1% predicted [92]. A population-based study of TCS reported to North American and European cancer registries found that patients treated with chemotherapy (with or without radiotherapy) in 1975 or later had a 1.6-fold higher risk of mortality (95% CI 1.25–2.01) (median follow-up: 10 years) due to respiratory diseases compared to the general population. The extent to which bleomycin-induced lung toxicity may have contributed to these excesses is not known.

## 7. Nephrotoxicity

Cisplatin damages the proximal and distal renal tubular epithelium and the renal collecting duct system, as well as the glomeruli at higher doses [98, 99]. Two long-term studies [100, 101] reported persistently decreased renal function in TCS for years after completion of treatment compared with baseline assessments. A Norwegian study [100] of 85 TC patients more than 10 years after treatment showed that renal function among TCS who received radiotherapy alone decreased by 8%, whereas survivors who had cisplatin-based chemotherapy had reductions of 14%. Cumulative cisplatin dose and age at treatment were both directly associated with long-term impairment of renal function (*P* < 0.05). A Danish investigation [101] of 34 TCS who received systemic chemotherapy with PVB (median dose of cisplatin: 583 mg/m^2^) reported that the glomerular filtration rate (GFR) decreased by a median of 18% during treatment. At a median follow-up of 65 months (range, 43 to 97 months), 38% of survivors had persistent renal dysfunction. Research in the general population has demonstrated a relationship between decreased GFR and the presence of microalbuminuria, leading to increased risks of CVD and all-cause mortality [102, 103]. Among long-term TCS, treatment-related nephrotoxicity may contribute to the reported increases in incident CVD events, including hypertension and MI [34, 35, 47]. To limit the severity of acute- and long-term renal damage, health care providers should administer hydration [104] and avoid nephrotoxic drugs [7] during cisplatin-based chemotherapy.

## 8. Hypogonadism

Orchiectomy, testicular dysgenesis syndrome, postorchiectomy chemotherapy or radiotherapy, and aging are the predominant causes of hypogonadism and premature hormonal aging in long-term TCS [105]. A Norwegian investigation [105] reported that 307 TCS treated between 1980 and 1994 had significantly increased risks of low testosterone as well as high-luteinizing hormone (LH) and follicle-stimulating hormone (FSH) levels after radiotherapy or chemotherapy at long-term follow-up. The degree of hypogonadism was directly related to the intensity of TC treatment [105–110]. A recent meta-analysis reported that both standard cisplatin-based chemotherapy (OR: 1.8) and infradiaphragmatic radiotherapy (OR: 1.6) significantly increased the risk of hypogonadism among TCS, as defined by total testosterone levels less than reference levels or use of testosterone replacement therapy, when compared to orchiectomy alone [110]. Hypogonadism may lead to reduced sexual functioning and well-being, fertility problems, muscle weakness, osteoporosis, loss of energy, and depression [111–117]. Further, hypogonadism is directly associated with the metabolic syndrome and CVD [29, 30, 32]. A recent multi-institutional cross-sectional study [118] reported that over one-third of North American TCS had hypogonadism at a median age of 38 years; in addition, hypogonadism was associated with increased CVD risk factors (i.e., dyslipidemia, hypertension, and diabetes), erectile dysfunction, and medication use for anxiety/depression (*P* < 0.05). Health care providers should regularly assess TCS for symptoms of hypogonadism and check hormonal status as clinically indicated. Clinical symptoms of hypogonadism should guide treatment decisions with testosterone replacement therapy [7]. Referral to endocrinologists for evaluation and management of difficult cases should also be considered.

## 9. Infertility

The association of infertility and TC is well established. Approximately 50% of patients with newly diagnosed TC have decreased sperm counts (<20 million/mL), low sperm motility indices (<40), and a high percentage of abnormal sperm cells (>80%) prior to initiation of any radiation or chemotherapy [119]. In a multicenter prospective study of 2318 TC patients in Germany and Austria [120], TC patients had significantly reduced spermatogenesis in their contralateral testicles confirmed histologically when compared to healthy subjects. The overall conception and paternity rates among long-term TCS with known intention to conceive a child after treatment completion range from 49% to 88% in several investigations (range of median follow-up: 7 to 12 years) [109, 121–123].

Radiotherapy can adversely affect reproductive function of TCS in the short-term [124, 125], since spermatogonia are the most sensitive germ cells to radiation treatment [126]. In the SWOG-8711 clinical trial of 207 patients with seminoma [124], sperm concentration reached a nadir 4 to 6 months after completion of radiotherapy, but returned to pretreatment level by 10 to 24 months after end of treatment. Similarly, Gandini et al. [127] reported that the sperm counts of TC patients reached a nadir at 6 months after radiation treatment, but 94% of patients recovered sperm counts by 2 years after end of radiotherapy. Higher dose of radiation is directly associated with longer recovery time for sperm concentration, and the use of testicular shielding devices significantly improves recovery of spermatogenesis [124]. A recent investigation of 1191 Norwegian TCS (median follow-up: 11 years) confirmed that radiotherapy had no significant long-term effects on sperm counts when compared to the surgery-only cohort [125].

In a retrospective study [128] of 178 TC patients treated with cisplatin-based chemotherapy in England, 64%, 24%, and 15% of patients who were normospermic, oligospermic and azoospermic, respectively, in the prechemotherapy period recovered normal spermatogenesis at least one year after chemotherapy completion. Prechemotherapy normospermia (HR 6.0), use of carboplatin versus cisplatin (HR 4.4), and noninclusion of a vinca alkaloid (HR 5.3) in chemotherapy regimen were significantly associated with normal recovery of sperm counts [128]. Cumulative dose of cisplatin-based chemotherapy is directly associated with infertility risk [112, 123, 125, 128]. In a multicenter investigation [123] of 316 Norwegian TCS (median follow-up: 12 years), 100%, 83%, and 76% of survivors self-reported achieving posttreatment paternity after 2, 3, and 4 cycles of standard cisplatin-based chemotherapy, respectively (*P*=0.022). However, sperm counts were not significantly related to number of cycles of chemotherapy in a limited cohort of patients for whom the results of semen analysis were available (*N* = 71) [123]. After a median follow-up of 10.6 years, another study [112] showed that TCS treated with high-dose chemotherapy (>850 mg cumulative cisplatin dose) had the lowest 15-year actuarial posttreatment paternity rate (48%) compared to 92% in the surveillance group and 60% in those treated with low-dose chemotherapy (≤850 mg cumulative cisplatin) (*P* < 0.001). Similarly, a recent investigation [125] reported that sperm counts and serum level of inhibin B were significantly lower in TCS treated with >850 mg cumulative cisplatin dose compared to those who had either surgery only or ≤850 mg cumulative cisplatin, whereas the serum FSH was significantly higher.

Among patients with stage II or III nonseminomatous germ cell tumor who have had a serologic complete response but have persistent enlarged retroperitoneal lymph nodes after cisplatin-based chemotherapy, RPLND is a standard treatment. To potentially avoid toxicities associated with cisplatin-based chemotherapy, RPLND is another treatment option for low-volume stage II nonseminomatous germ cell tumor with normal β-hCG and AFP levels after orchiectomy [62]. Injury to the retroperitoneal postganglionic sympathetic nerves during RPLND may result in retrograde ejaculation [126], leading to inability to conceive without use of cryopreserved sperm. The rate of retrograde ejaculation ranges from 1 to 9% after primary RPLND [129–131], 11% to 29% after nerve-sparing postchemotherapy RPLND [130, 132–134], and 75% after full bilateral postchemotherapy RPLND [135].

Health care providers should address the possibility of infertility and discuss fertility preservation options with TC patients as detailed by the American Society of Clinical Oncology Clinical Practice Guideline for fertility preservation for patients with cancer [136]. If clinically indicated, referral to appropriate reproductive specialists should be considered [136]. Sperm cryopreservation is a standard fertility preservation practice [136] that may be offered to interested TC patients undergoing treatment.

## 10. Avascular Necrosis

Avascular necrosis commonly affects the femoral head, often bilaterally [137], with an incidence of approximately 1-2% in long-term TCS treated with cisplatin-based chemotherapy [137, 138]. The etiology for avascular necrosis is multifactorial [7] but likely to be partially attributable to corticosteroids used as antiemetics during TC treatment [137–140]. Bleomycin and vinblastine have also been hypothesized as causative agents in a case of avascular necrosis in one TCS who did not receive corticosteroids during chemotherapy [141]. Health care providers should review with TC patients who receive high-dose corticosteroids the potential risk of avascular necrosis. For any long-term TCS who develops early symptoms suggestive of avascular necrosis, including decreased hip motion and/or limp, prompt evaluation with plain radiograph or MRI is critical [7].

## 11. Cognitive Impairment

The underlying mechanisms of chemotherapy-related cognitive impairment have not been elucidated. Several neuroimaging studies of breast cancer survivors have reported that white matter activation patterns involved in cognitive functioning are altered after chemotherapy [142–144], likely due to neurotoxic effects [145]. A study [146] of 66 TC patients suggested that cortisol levels prior to chemotherapy may be a predictor of later cognitive complaints. The prevalence of cognitive impairment in men with newly diagnosed TC before receipt of any chemotherapy ranges from 46% to 58% and is significantly higher than expected in the healthy normal population (*P* < 0.01) [146, 147]. A prospective clinical trial [88] of 666 patients with metastatic TC in Europe showed that cognitive function decreased at 3 months after chemotherapy, though not at the level of clinical relevance but recovered to baseline values at 2 years for most patients, with 19% still having worsened cognitive function at that time. However, the association of cisplatin-based chemotherapy with cognitive impairment in TCS remains unclear. Whereas three studies [148–150] of TCS (*N* = 70–112; median follow-up: 1–3 years) reported no significant differences in performance on cognitive tests between TC treatment groups (i.e., surgery only versus chemotherapy), two investigations [151, 152] reported increased risks of cognitive impairment after chemotherapy. Among 1173 TCS with a median follow-up of 9 years, Skoogh et al. [151] reported a 2-fold increased risk (95% CI 1.3–3.1) of long-term compromised speech in survivors who completed five or more cycles of cisplatin-based chemotherapy compared to those who received no chemotherapy. Similarly, a single institutional prospective study [152] of TC patients after orchiectomy who either received adjuvant chemotherapy (*N* = 55) or no additional treatment (*N* = 14) reported that chemotherapy was significantly associated with cognitive decline with a dose-response relationship observed at 12 months (surveillance group: 0%; 2-3 cycles of chemotherapy: 52%; and 4–7 cycles of chemotherapy: 67%). Although the extent to which cisplatin-based chemotherapy may have negative effects on long-term cognitive function in TCS is unclear, cognitive complaints among long-term survivors are common and independent of treatment modality [88, 148–151]. These subjective complaints may reflect the effects of anxiety and depression, which are prevalent in TCS [148]. The first step in managing cognitive complaints may include managing specific stressors by implementing effective coping strategies.

## 12. Anxiety/Depression

A Norwegian study [153] reported a significantly higher prevalence of a Hospital Anxiety and Depression Scale- (HADS-) defined anxiety disorder among TCS (mean follow-up time: 11.3 years) compared to age-adjusted men from the general population (19.2% versus 13.5%, *P* < 0.001). Young age, peripheral neuropathy, economic difficulties, excess alcohol use, sexual concerns, and prior treatment for mental illness were significantly associated with HADS-defined anxiety disorder [153]. A recent investigation [154] showed that the prevalence of clinically significant anxiety among TCS (mean: 11.6 years after diagnosis) in Germany was 6.1%. Anxiety was significantly associated with younger age at diagnosis and shorter time since diagnosis in multivariate analyses. Prior studies [153–156] reported that the prevalence of depression among TCS ranges from 7.9% to 20%, but the extent to which TCS may experience significantly more depressive orders compared to the general population is uncertain. Feeling helpless/hopeless [156], lower social support [156], a higher number of physical symptoms [154], and having children [154] were reported to be significantly associated with higher levels of depression.

## 13. Fatigue

Chronic fatigue, defined as symptoms with a duration of ≥6 months, is a common and distressing cancer-related adverse effect [157]. The prevalence of chronic cancer-related fatigue among Norwegian TCS was significantly higher compared to age-matched men in the general population (17.1% versus 9.7%) [158]. A recent longitudinal investigation [159] of 812 TCS treated between 1980 and 1994 in Norway reported that the prevalence of chronic fatigue increased from 15% at survey I (1998–2002) to 27% at survey II (2007-2008) (*P* < 0.001). Several factors were significantly associated with chronic fatigue in this study: [159] high level of neuropathy, Raynaud-like phenomena, testosterone level in the lowest quartile, low level of physical activity, as well as higher levels of anxiety and depression. Health care professionals should consider exercise and psychological interventions for early prevention and treatment of chronic fatigue among TCS. A recent meta-analysis of cancer survivors [160] reported that exercise and psychological interventions are effective for reducing cancer-related fatigue during and after cancer treatment and significantly more effective than available pharmaceutical options.

## 14. Adverse Health Outcomes

**Figure 2:**
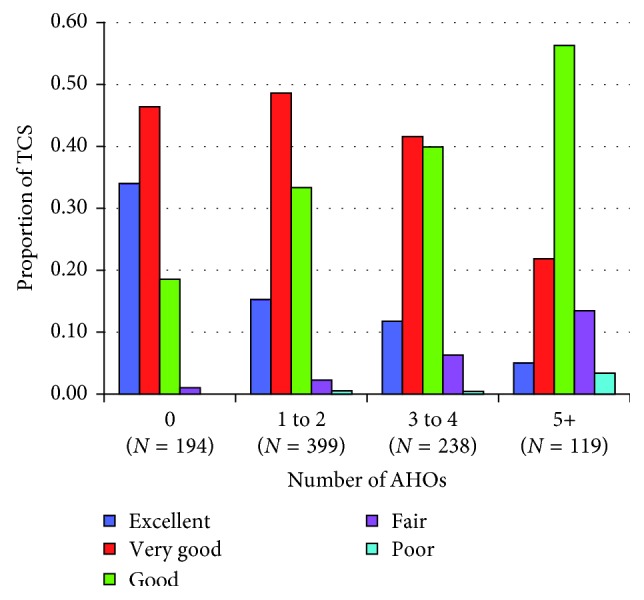
Proportion of testicular cancer survivors (TCS) with excellent, very good, good, fair, and poor self-reported health by number of adverse health outcomes (AHOs). *P* value for association of number of AHOs with self-reported health was <0.01 (Mantel 1 df chi-square test of trend). Self-reported health was not indicated by one participant with 1-2 AHOs and one participant with 3-4 AHOs. ^∗^Adapted with permission from Fung et al. [40] (Figure 1).

To develop risk-stratified, evidence-based follow-up recommendations for TCS, characterization of long-term adverse health outcomes (AHOs) is critical. A recent multi-institutional investigation [40] of 952 North American TCS examined the type and prevalence of AHOs after chemotherapy with four cycles of EP (EPX4) or three or four cycles of BEP (BEPX3/BEPX4) (Table 3). At a median age of 37 years, more than one-third of survivors reported three or more AHOs with similar prevalence and type after EPX4 and BEPX3, except for Raynaud phenomenon (11.6% versus 21.4%; *P* < 0.01), peripheral neuropathy (29.2% versus 21.4%; *P*=0.02), and obesity (25.5% versus 33.0%; *P*=0.04). The type and prevalence of AHOs after BEPX4 were largely similar to EPX4 and BEPX3. Increasing age at clinical evaluation, current tobacco use, and nonmarried status were associated with increased numbers of AHOs, whereas weekly vigorous physical activity was protective (*P* < 0.05). Self-reported health was excellent/very good in approximately 60% of TCS, but this proportion decreased as number of AHOs increased (*P* < 0.001) (Figure 2).

## 15. Conclusions

Due to their young age at diagnosis, long-term survival, and current use of largely homogeneous therapies, TCS comprise an ideal cohort for adult-onset cancer survivorship research [161]. Moreover, these patients now comprise approximately 4% of all male cancer survivors [162].  Table 4 summarizes major research priorities for TC survivors set forth at an international consensus conference [161]. An overarching recommendation was the development of longitudinal cohort studies to evaluate the life-long burden and latency trends of medical and psychosocial morbidities by category of treatment. TC is relatively unique among cancer types in that it provides for the ready availability of a “comparison group” cured with surgery only without the confounding effects of cytotoxic treatment, with which to compare the late effects of radiotherapy and chemotherapy. Moreover, study of the surgery-only group itself is informative and presents a unique opportunity to study the long-term history of a cancer cured without cytotoxic therapy, including any inherently preprogrammed development of adverse metabolic and other outcomes. This type of proposed cohort investigation, which gathers comprehensive exposure and outcome data, can provide the basis for identifying predictors of AHOs, either singly or jointly, for the eventual development of preventive and interventional measures. An important goal not only for TCS, but for cancer survivors in general, is the identification of genetic variants that predispose to the development of acute and long-term treatment toxicities. This elucidation of etiopathogenetic pathways provides another step towards developing targeted prevention and intervention strategies to optimize risk-based care, minimize chronic morbidities, and improve patients' quality of life.

## Figures and Tables

**Table 1:** Prevalence of cardiovascular disease risk factors in chemotherapy-treated patients in select studies since 2000.

						Prevalence of cardiovascular risk factors versus Controls^b^ (%)				
Author (year)	*N* ^a^	Treatment dates	Median length of follow-up (range)	Median age at follow-up (range)	Control group (*N*)	HTN (definition)	Increased lipids^c^ (definition)	DM (definition)	Obesity (definition)	Metabolic syndrome (definition)
Meinardi et al. (2000) [16]	62	Before 1987	14 y (10–20 y)	42 y (30–50 y)	Stage I TC (40)	39 versus 13 (SBP > 150 mmHg, DBP > 95 mmHg)	79 versus 53 (TC ≥ 201 mg/dL)	NA	21 versus 28^d^ (BMI > 27.8)	NA
Strumberg et al. (2002) [30]	32	1977–1981	15 y (13–17 y)	40 y (30–59 y)	None	25 versus NA (DBP > 95 mmHg)	81 versus NA (TC ≥ 200 mg/dL)	NA	48 versus NA (BMI ≥ 25)	NA
Huddart et al. (2003) [35]	390	1982–1992	10 y (0–20 y)	41 y (23–72 y)	Stage I TC (242)	13 versus 9^d^ (antihypertension medication)	1 versus 2^d^ (lipid-lowering medication)	NA	NA	NA
Nuver et al. (2004) [14]	90	1988–1999	7 y (NA)	37 y (20–65 y)	Stage I TC (44) and healthy patients (47)	22 versus 23 versus 11^d^ (SBP ≥ 135 mmHg, DBP ≥ 85 mmHg)	71 versus 59 versus 45^e^ (LDL > 131 mg/dL)	NA	22 versus 27 versus 11^e^ (BMI ≥ 30)	NA
Nuver et al. (2005) [36]	86	1988–1999	7 y (3–13 y)	37 y (20–65 y)	Stage I TC (44) and healthy patients (47)	NA	NA	NA	NA	26 versus 36 versus 9^e^ (≥3 factors per NCEP definition [163])
Sagstuen et al. (2005) [37]	500	1980–1994	11 y (4–22 y)	44 y (23–75 y)	Surgery-only^f^ (242)^g^	50 versus 39 (SBP ≥ 140 mmHg or DBP ≥ 90 mmHg or antihypertension medication)	NA	NA	18 versus 13^h^ (BMI ≥ 30)	NA
Haugnes et al. (2007) [33]	464	1980–1994	11 y (5–22 y)	43 y (15–52 y)	Surgery-only^f^ (225) and healthy population (1150)	45 versus 34 versus 50^i^ (SBP ≥ 140 mmHg or DBP ≥ 90 mmHg or antihypertension medication)	68 versus 67 versus 84^d^ (TC ≥ 201 mg/dL)	3 versus 2 versus 3^d^ (by patient self-report)	18 versus 13 versus 21^d^ (BMI ≥ 30)	9 versus 7 versus 15^h^ (≥3 factors per NCEP definition [163])
Haugnes et al. (2010) [34]	364	1980–1994	19 y (13–28 y)	49 y (31–69 y)	Surgery-only^f^ (206) and healthy population (990)	26 versus 12 versus 13 (antihypertension medication)	14 versus 14 versus 9^e^ (lipid-lowering medication)	5 versus 4 versus 4^d^ (by patient self-report or fasting glucose ≥ 198 mg/dL)	17 versus 19 versus 23^d^ (BMI ≥ 30)	NA
Willemse et al. (2013) [38]	194^j^	1977–2008	7.8 y (0.1–30 y)	39.6 y (18–70 y)	Surgery-only (57) and healthy population (360)	29 versus 14 versus 22.5^k^ (per NCEP definition [163])	NA	NA	29 versus 18 versus 19^k^ (per NCEP definition [163])	16 versus 9 versus 8^k^ (per NCEP definition [163])
de Haas et al. (2013) [39]	173	1977–2004	5 y (3–20 y)	37 y (19–59 y)	Healthy population (1085)	59 versus NA (SBP ≥ 130 mmHg or DBP ≥ 85 mmHg or antihypertension medication) (AHA/NHLBI definition)	44 versus NA (HDL <1.03 mmol/l or lipid-lowering medication) (AHA/NHLBI definition)	14 versus NA (fasting glucose ≥5.6 mmol/l or medication) (AHA/NHLBI definition)	17 versus NA (waist circumference ≥ 102 cm) (AHA/NHLBI definition)	25 versus NA (≥3 factors per AHA/NHLBI definition)
Fung et al. (2017) [40]	952	1979–2015	4.3 y (1.0–29.9)	37 y (19–68 y)	NHANES matched controls (952)	16.8 versus 19.4^d^ ever diagnosed with high blood pressure and current use of antihypertension medication	10.5 versus NA^l^ current use of cholesterol lowering medication	3.1 versus NA^l^ diabetes requiring insulin or diabetes requiring medication	31.3 versus 35.4^k^ BMI ≥ 30	NA

^∗^Adapted with permission from Feldman et al. [9] (Table 2). AHA: American Heart Association; BMI: body mass index; DBP: diastolic blood pressure; DM: diabetes mellitus; NHLBI: National Heart, Lung, and Blood Institute; NHANES: National Health and Nutrition Examination Survey; TC: testicular cancer; HTN: hypertension; LDL: low-density lipoprotein; NA: not available; NCEP: National Cholesterol Education Program; SBP: systolic blood pressure; TC: total cholesterol. (a) *N* varies slightly for individual factors due to missing data in papers for some variables. (b) Definitions of individual factors vary by study. Comparisons of chemotherapy group to controls significant unless otherwise stated; percentages vary slightly due to missing data for individual factors. (c) Cholesterol and fasting glucose values in definitions were converted from mml/L to mg/dL, where necessary for uniformity. (d) Not significant. (e) Significant versus healthy population controls but not versus surgery patients. (f) Includes both orchiectomy and primary retroperitoneal lymph node dissection patients. (g) A healthy population control group was also included in this study, but prevalence rates of cardiovascular risk factors were not reported for this control group, and therefore these data are not included in the table. (h) Significant only for patients who received >850 mg of cisplatin. (i) Significant versus surgery patients but not versus healthy controls. (j) 20 patients received carboplatin and 174 patients received combination chemotherapy. (k) Significant for patients who received combination chemotherapy compared to healthy population. (l) Significance versus controls not tested.

**Table 2:** Relative risks of second malignant neoplasms (SMN) in testicular cancer survivors.

	No. of patients	Calendar years of testicular cancer diagnosis	Duration of follow-up (years)	Treatment	Obs.	RR	(95% CI)
*Study populations* ^a^							
*All SMNs*							
Norwegian radium hospital [66]	2006	1952–1990	Mean = 12.5	Any	153^b^	1.7	1.4–1.9
				RT	130	1.6	1.3–1.9
				CT	4	1.3	0.4–3.4
				RT + CT	15	3.5	2.0–5.8
Fourteen population-based tumor registries in Europe and North America [55]	40,576	1943–2001	Mean = 11.3	Any	1694	1.9	1.8–2.1
				RT	892	2.0	1.9–2.2
				CT	35	1.8	1.3–2.5
				RT + CT	25	2.9	1.9–4.2
Thirteen International Cancer Registries [164]	29,511	1943–2000	Median = 8.3	Any	1811^c^	1.7	1.6–1.7
Netherlands testicular cancer survivor cohort [46]	2707	1965–1995	Median = 17.6	Any	270^d^	1.7	1.5–1.9
				RT	199	1.7	1.5–2.0
				CT	23	1.4	0.9–2.1
				RT + CT	29	3.0	2.0–4.4
				SDRT	N/A	2.6^g^	1.7–4.0
				SDRT + MRT	N/A	3.6^g^	2.1–6.0
				PVB/BEP	N/A	2.1^g^	1.4–3.1
				SDRT (26–35 Gy)	N/A	2.3^g^	1.5–3.6
				SDRT (40–50 Gy)	N/A	3.2^g^	2.1–5.1
Swedish family cancer database [165]	5533	1980–2006	N/A	Any	274^e^	2.0	1.8–2.2
*Second solid cancers*	12,691	1980–2008	Median = 7.0	Initial surgery only	99	0.9	0.8–1.1
Sixteen population-based registries within the SEER program [67]				Initial CT (no RT)	111^f^	1.4	1.2–1.7
*Therapy-associated leukemia*							
Nested case-control study of leukemia in 8 population-based tumor registries in Europe and North America [56]	18,567	1970–1993	N/A	No RT/CT	4	1.0	—
				RT	22	3.1	0.7–2.2
				CT	8	5.0	1.1–40
				RT + CT	2	5.1	0.5–28

^∗^Adapted with permission from *Fung* et al. *J Natl Compr Canc Netw 2012; 10:545-56* (Table 2). RR: relative risk; CI: confidence interval; Obs.: observed number of cases; RT: any radiation treatment; CT: chemotherapy; IDRT: infradiaphragmatic radiation; SDRT: supradiaphragmatic radiation; MRT: mediastinal radiation; PVB: cisplatin, vinblastine, bleomycin; BEP: bleomycin, etoposide, cisplatin; N/A: not available (data not provided). (a) There was overlap in the cancer registries included in the cohort studies by Richiardi et al. [164] and Travis et al. [55], with the following countries contributing patients to both studies: Denmark, Finland, Norway, and Sweden; (b) six cases of leukemia were observed with a RR of 1.9 (95% CI: 0.7–4.1); (c) thirty-eight cases of myeloid leukemia were observed with a RR of 3.6 (95% CI: 2.6–5.0); thirteen cases of lymphoid leukemia were observed with a RR of 1.0 (95% CI: 0.5–1.7); twenty-three cases of other types of leukemia were observed with a RR of 3.5 (95% CI: 2.2–5.2); (d) six cases of leukemia were observed with a RR of 1.6 (95% CI: 0.6–3.5); (e) hazard ratios are shown, with the referent group consisting of patients treated with surgery alone (HR = 1.0). Twelve cases of leukemia were observed with a RR of 3.8 (95% CI: 2.0–6.7); (f) significantly increased risks occurred for cancers of the kidney (SIR = 3.4; 95% CI 1.8–5.7; *n* = 13); thyroid (SIR = 4.4; 95% CI: 2.2–7.9; *n* = 11); and soft tissue (SIR = 7.5; 95% CI: 3.6–13.8; *n* = 10).

**Table 3:** Numbers and types of self-reported adverse health outcomes among 952 cisplatin-treated germ cell tumor survivors in North America^∗^.

Adverse health outcomes (AHOs)	Total patients (*N* = 952) *N* (%)	Treatment regimen		
		EP (4 cycles) (*N* = 294) *N* (%)	BEP (3 cycles) (*N* = 364) *N* (%)	BEP (4 cycles) (*N* = 170) *N* (%)
*Total number of AHOs*				
Median (range)	2 (0–11)	2 (0–9)	2 (0–11)	2 (0–10)
0	194 (20.4)	64 (21.8)	83 (22.8)	25 (14.7)
1	209 (21.9)	68 (23.1)	71 (19.5)	42 (24.7)
2	191 (20.1)	61 (20.8)	82 (22.5)	27 (15.9)
3	143 (15.0)	48 (16.3)	48 (13.2)	25 (14.7)
4	96 (10.1)	28 (9.5)	38 (10.4)	18 (10.6)
5 or more	119 (12.5)	25 (8.5)	42 (11.5)	33 (19.4)
*Type of AHOs* ^†^				
Yes	353 (37.1)	104 (35.4)	130 (35.7)	65 (38.2)
No^‡^	599 (62.9)	190 (64.6)	234 (64.3)	105 (61.8)
*Hearing impairment* ^§^				
Yes	300 (31.5)	95 (32.3)	109 (30.0)	56 (33.0)
No	652 (68.5)	199 (67.7)	255 (70.0)	114 (67.0)
*Peripheral neuropathy* ^ǁ^				
Yes	257 (27.0)	86 (29.2)	78 (21.4)	54 (31.8)
No	695 (73.0)	208 (70.8)	286 (78.6)	116 (68.2)
*Peripheral neuropathy plus tinnitus and/or hearing issue*				
Yes	156 (16.4)	49 (16.7)	48 (13.2)	31 (18.2)
No	796 (83.6)	245 (83.3)	316 (86.8)	139 (81.8)
*Hypertension and on prescription medication*				
Yes	110 (11.6)	35 (11.9)	45 (12.4)	15 (8.8)
No^¶^	842 (88.4)	259 (88.1)	319 (87.6)	155 (91.2)
*Hypercholesterolemia and on prescription medication*				
Yes	100 (10.5)	32 (10.9)	31 (8.5)	20 (11.8)
No^∗∗^	852 (89.5)	262 (89.1)	333 (91.5)	150 (88.2)
*Cardiovascular disease* ^††^				
Yes	14 (1.5)	4 (1.4)	4 (1.1)	2 (1.2)
No^‡‡^	938 (98.5)	290 (98.6)	360 (98.9)	168 (98.8)
*Raynaud phenomenon*				
Yes	178 (18.7)	34 (11.6)	78 (21.4)	49 (28.8)
No^§§^	774 (81.3)	260 (88.4)	286 (78.6)	121 (71.2)
*Peripheral vascular disease*				
Yes	29 (3.0)	5 (1.7)	8 (2.2)	10 (5.9)
No^ǁǁ^	923 (97.0)	289 (98.3)	356 (97.8)	160 (94.1)
*Thromboembolic disease* ^¶¶^				
Yes	5 (0.5)	0	0	4 (2.4)
No	947 (99.5)	294 (100)	364 (100)	166 (97.6)
*Renal disease*				
Yes	25 (2.6)	7 (2.4)	6 (1.6)	7 (4.1)
No^∗∗∗^	927 (97.4)	287 (97.6)	358 (98.4)	163 (95.9)
*Diabetes and on prescription medication* ^†††^				
Yes	30 (3.1)	9 (3.1)	10 (2.7)	3 (1.8)
No	922 (96.9)	285 (96.9)	354 (97.3)	167 (98.2)
*Benign thyroid disease*				
Yes	23 (2.4)	6 (2.0)	9 (2.5)	5 (2.9)
No^‡‡‡^	929 (97.6)	288 (98.0)	355 (97.5)	165 (97.1)
*Problems with balance/vertigo/dizziness* ^§§§^				
Yes	89 (9.3)	26 (8.8)	37 (10.2)	16 (9.4)
No	863 (90.7)	268 (91.2)	327 (89.8)	154 (90.6)
*Hypogonadism with testosterone therapy* ^ǁǁǁ^				
Yes	93 (9.9)	25 (8.6)	37 (10.3)	16 (9.5)
No	851 (90.1)	267 (91.4)	323 (89.7)	152 (90.5)
*Erectile dysfunction*				
Yes	115 (12.1)	28 (9.5)	39 (10.7)	34 (20.0)
No^¶¶¶^	837 (87.9)	266 (90.5)	325 (89.3)	136 (80.0)
*Psychotropic prescription medication for anxiety and/or depression* ^∗∗∗∗^				
Yes	99 (10.4)	34 (11.6)	27 (7.4)	20 (11.8)
No	853 (89.6)	260 (88.4)	337 (92.6)	150 (88.2)

^∗^Adapted with permission from Fung et al. [40] (Table 3). BEP: bleomycin, etoposide, cisplatin; CAD: coronary artery disease; EP: etoposide, cisplatin; MI: myocardial infarction. ^†^*P* values are derived from the chi-square test comparing the proportions of AHOs reported by TCS in the EPX4 and BEPX3 treatment groups. Except for Raynaud phenomenon (*P* < 0.01) and peripheral neuropathy (*P*=0.02), the *P* values for all other AHOs were >0.05; category includes 3 participants for whom this outcome was not stated; among all 952 participants, 270 (28.4%) reported problems hearing words, sounds, or language in crowds, 13 (1.4%) required hearing aid, and 2 (0.2%) had complete deafness (questions derived from the hearing handicap inventory by Ventry and Weinstein) [166]; 109 (11.4%) had “quite a bit” or “very much” difficulty hearing and 75 (7.9%) had “quite a bit” or “very much” reduced hearing (EORTC-CIPN20 and SCIN) ([167, 168]). Category includes 48 participants for whom this outcome was not stated; among all 952 participants, the number of patients reporting “quite a bit” or “very much” to the following questions are as follows: 123 (12.9%) tingling fingers or hands, 167 (17.5%) tingling toes or feet, 121 (12.7%) numbness in fingers or hands, 161 (16.9%) numbness in toes or feet, 34 (3.6%) shooting/burning pain in fingers or hands, 70 (7.4%) shooting/burning pain in toes or feet (EORTC-CIPN20) [167]; 134 (14.1%) pain and tingling in toes or feet, and 86 (9.0%) pain and tingling in hands or fingers (SCIN) [168]. Category includes 16 participants for whom this outcome was not stated; category includes 11 participants for whom this outcome was not stated; ^∗∗^ category includes 3 participants for whom this outcome was not stated; ^††^includes coronary artery disease, heart failure, and cerebrovascular disease (categories not mutually exclusive, and each category was counted as one AHO). Among all participants, 7 (0.7%) reported coronary artery disease (3 occurrences for coronary artery disease, 5 occurrences of angioplasty or stent, and 5 occurrences of heart attack or myocardial infarction); 1 patient reported heart failure; and 10 (1.0%) reported cerebrovascular disease (6 occurrences of transient ischemic attacks, 4 occurrences of stroke, and 1 occurrence of carotid artery surgery); ^‡‡^category includes 21 participants for whom this outcome was not stated; ^§§^category includes 12 participants for whom this outcome was not stated; ^ǁǁ^category includes 19 participants for whom this outcome was not stated; ^¶¶^deep vein thrombosis (DVT) and pulmonary embolism (PE) developed simultaneously in 3 participants and was counted as one thromboembolic event for each. The remaining 2 participants reported DVT only. Category includes 19 participants for whom this outcome was not stated; ^∗∗∗^category includes 26 participants for whom this outcome was not stated; ^†††^among all participants, 13 (1.4%) and 22 (2.3%) reported use of insulin and oral antiglycemic agents, respectively (categories not mutually exclusive). Category includes 15 participants for whom this outcome was not stated; ^‡‡‡^category includes 19 participants for whom this outcome was not stated; ^§§§^of the 89 patients, 47 reported persistent dizziness or vertigo and 63 reported symptoms of dizziness when standing up (categories not mutually exclusive). Category includes 40 participants for whom this outcome was not stated; ^ǁǁǁ^eight participants who underwent bilateral orchiectomy were excluded from this category; ^¶¶¶^category include 7 participants for whom this outcome was not stated; ^∗∗∗∗^participants could report more than one psychotropic medication. Psychotropic medications used by the 99 participants include aripirazole (*n* =2), alprazolam (*n* = 5), amphetamine-dextroamphetamine (*n* = 9), bupropion (*n* = 10), buspirone (*n* = 1), citalopram (*n* = 6), clonazepam (*n* = 8), desvenlafaxine (*n* = 1), diazepam (*n* = 1), duloxetine (*n* = 7), escitalopram (*n* = 16), fluvoxamine (*n* = 1), fluoxetine (*n* = 4), hydroxyzine (*n* = 1), lisdexamfetamine (*n* = 4), lorazepam (*n* = 6), methylphenidate (*n* = 5), nortriptyline (*n* = 2), olanzapine (*n* = 2), paroxetine (*n* = 7), trazodone (*n* = 5), sertraline (*n* = 11), and venlafaxine (*n* = 7).

**Table 4:** Summary of major research recommendations: late effects of testicular cancer and its treatment.

(1) *Overarching recommendation: lifelong follow-up of all testicular cancer survivors (TCS)*
(i) Integrate observational and analytic epidemiologic studies with molecular and genetic approaches to ascertain the risk of emerging toxicities and to understand the evolution of known late effects, especially with the aging of TCS.
(ii) Evaluate the influence of race and socioeconomic status (SES) on the late effects of TC and its treatment.
(iii) Characterize long-term tissue deposition of platinum (sites and reactivity), serum levels, and correlation with late effects.
(iv) Evaluate the life-long burden of medical and psychosocial morbidity by treatment.
(v) Utilize research findings to establish evidence-based, risk-adapted, long-term follow-up care.
(2) *Specific recommendations*
(i) Second malignant neoplasms (SMN) and late relapses
(a) Determine the effect of reductions in field size and dose of radiotherapy, along with the use of carboplatin as adjuvant therapy in seminoma patients, on the risk of SMN.
(b) Examine relation between platinum-based chemotherapy and site-specific risk of solid tumors, the associated temporal patterns, and the influence of age at exposure and attained age.
(c) Compare risk of SMN in TCS managed with surgery alone to cancer incidence in the general male population.
(d) Examine delaying influence of platinum-based chemotherapy (and duration and magnitude of effect) on development of contralateral testicular cancer.
(e) Characterize the evolution of cured testicular cancer, in particular, the molecular underpinnings of late recurrences.
(ii) Cardiovascular disease (CVD)
(a) Evaluate the contributions and interactions of subclinical hypogonadism, platinum-based chemotherapy, radiotherapy, lifestyle factors (diet, tobacco use, and physical activity), body mass index, family history of CVD, race, socioeconomic status, abnormal laboratory values, and genetic modifiers.
(b) Develop comprehensive risk prediction models, considering the above variables, to stratify TCS into risk groups in order to customize follow-up strategies and develop evidence-based interventions.
(iii) Neurotoxicity
(a) Evaluate evolution of neurotoxicity across TCS lifespan, role of genetic modifiers, and extent to which symptoms impact on work ability and quality of life.
(iv) Nephrotoxicity
(a) Determine whether the natural decline in renal function associated with aging is accelerated in TCS, any influence of low-level platinum exposure, and the impact of decreased GFR on CVD and all-cause mortality.
(b) Determine the incidence of hypomagnesemia, together with the role of modifying factors and resultant medical consequences, in long-term TCS.
(v) Hypogonadism and decreased fertility
(a) Address the incidence, course, and clinical effects of subclinical hypogonadism.
(b) Evaluate effect of all levels of gonadal dysfunction in TCS on CVD, premature aging, fatigue, osteoporosis, mental health, quality of life, and sexuality.
(vi) Pulmonary function
(a) Examine role of platinum compounds on long-term pulmonary damage in TCS, and interactions with other influences, including bleomycin, tobacco use, and occupational risk factors.
(vii) Psychosocial effects
(a) Identify prevalence and predictors of depression, cancer-related anxiety, fatigue, infertility-related distress, problems with sexuality and paired relationships, and posttraumatic growth.
(b) Examine the impact of different cultural backgrounds on posttreatment quality of life.
(c) Evaluate TCS work ability throughout life.
(d) Determine whether normal age-related declines in cognitive function are accelerated in TCS.
(3) *Interventions*
(i) Conduct targeted intervention trials aimed at promoting smoking cessation, healthy dietary habits, and an increase in physical activity.
(ii) Evaluate the role of information and communication technologies in promoting a healthy lifestyle among TCS.
(iii) Consider randomized, pharmacologic intervention trials among TCS with biochemical parameters approaching threshold values to avoid accelerated development into treatment-requiring CVD.
(iv) Determine optimal schedule of testosterone replacement therapy among TCS with clinical hypogonadism.
(v) Consider screening strategies for selected SMN.
(4) *Genetic and molecular considerations*
(i) Evaluate genetic risk factors (identified in the general male population) as modifiers for all late effects in TCS, in particular, CVD, SMN, neurotoxicity, nephrotoxicity, hypogonadism, and psychosocial effects.
(ii) Investigate the role of genome-wide association studies, epigenetics, mitochrondrial DNA, microRNA, proteomics and related approaches in identifying genetic variants that contribute to the late effects of treatment.
(iii) Develop standardized procedures for biospecimen collection to support genetic and molecular studies, as reviewed previously.
(5) *Risk prediction models*
(i) Develop comprehensive risk prediction models that incorporate genetic modifiers of late sequelae.

^∗^Adapted with permission from Travis et al. [161] (Table 2).
